# Michigan Dental Association

**Published:** 1892-12

**Authors:** 


### PROCEEDINGS.

### Michigan Dental Association.

THE thirty-seventh annual meeting of the Michigan Dental Association was called to order by the President, Dr. Henry C. Corns, at the City Hall in the City of Saginaw, on the morning of Thursday, June 2d, 1892.

After the divine blessing had been invoked by the Rev. Dr. Warren, the Association listened to an address of welcome by Mr. C. S. Smith, of Saginaw, the Hon. W. S. Linton, Mayor of the city, who was to have delivered the address being called away from home. Mr. Smith, on behalf of the citizens of Saginaw, extended to the members of the Association the hospitality and freedom of the city, and was assured that our stay would be one of pleasure and profit. The President responded in a few well chosen remarks, after which an opportunity was given for the members present to pay their annual dues.

There being nothing further to come before the meeting at this time, a motion to adjourn was carried.

### THURSDAY AFTERNOON, JUNE 2d.

Meeting was called to order by the President. The minutes of the preceding meeting were read and approved. The President's address was received in a hearty manner by the Association.

On motion of Dr. Jackson, of Ann Arbor, seconded by Dr. Alden, a vote of thanks was extended President Corns for his excellent address.

Dr. Whiting read his paper on, "Dentistry in 1840."

Dr. Douglas, related his experience in filling teeth before the introduction of rubberdam, by use of napkins, etc., he showed several teeth filled with soft gold as far back as 1850. The doctor had for examination operative instruments made at that time, the work of his own skill.

The Association then listened to a well worded and pointed paper by Dr. Morgan, of Saginaw, on "Ether as an Anæsthetic." Dr. Douglas opened the discussion, citing several cases which he had to deal with in the administration of chloroform and compared the relative merits of the two anæsthetics.

Dr. Essig said he had excellent results from Nitro-glycerine for resuscitating patients; also thought the cone the best appliance for the distribution of ether.

Dr. Jackson, of Ann Arbor, was of the impression that death from chloroform was, in a great many cases, the result of carelessness on the part of the administrator of the anæsthetic, not enough attention being given to the details of such an operation.

Dr. Pelton, of Au Sable, thought that we should not administer an anæsthetic when the patient had heart trouble to any degree, and impressed this fact: that the welfare of patients should come before that of the operator.

Dr. Morgan closed the discussion by saying he had rather give ether in cases of apparent heart trouble than chloroform; that, in post mortem examinations, there has never been found any heart trouble from the administration of ether.

Dr. C. B. Blackmarr, of Jackson, read a paper on "Business and Professional Methods with our Patient's," the discussion of which was left over for the next session.

A motion to adjourn was carried.

### THURSDAY EVENING, JUNE 2d.

Dr. E. C. Moore, who opened the discussion of Dr. Blackmarr's paper, agreed so thoroughly with the essayist that he had no points to add nor faults to find.

Dr. Douglas read a paper on "Difficult Prosthesis."

Dr. Whiting had seen one of the cases and spoke of the difficulty which attended it.

Dr. Long, in speaking of Dr. Douglass' paper, dwelt upon this one point, "The Determination to Overcome every Difficulty." Dr. Douglass having had to use his own mechanical judgment in the cases cited.

Dr. Sweetnam, of Manistee, read a paper on "How to Practice Dentistry Successfully."

The discussion was opened by Dr. G. E. Sanders, of Saginaw, who dwelt upon the education of our patients and urged the necessity upon the dentists of keeping abreast with the times by using only the latest methods for saving the teeth; he advocated dental literature being distributed among the medium classes for their edification, also the exhibition of kindness to children, who should have our most sincere sympathies, and thereby gain their confidence; and a thorough understanding of our business, and, by our failures, to bring about success and a better feeling mong the profession to supplant that feeling of jealousy and rejoice in the success of others; no insinuation should poison the mind of our brother's patient; build up your business on the science of dentistry from your own knowledge, and not from the failure of others.

Dr. Long, of Monroe, thought the paper covered broad ground; was pleased with it. He would advocate in the dentist, sobriety, honesty, cleanliness, kindness, truthfulness, industry, he should bear in mind that the patient's time is valuable as well as ours; a dentist should take a professional standing and live up to it.

Dr. Pelton, of Au Sable; was very much impressed with the paper, emphasized the one point with reference to the lazy man, advocated keeping good hours, always be ready to proceed with everything, and crowd his work instead of the work crowding him, should be strictly honest with all his patients, more especially children.

Dr. Jackson, of Ann Arbor, related his experience as regards honesty in practice, by which he gained the confidence of a child by telling the truth, success in life is "doing the greatest amount in life." "Good work will always command good prices."

Adjourned to Friday morning.

### FRIDAY MORNING, JUNE 3d.

Meeting was called to order. Minutes of the previous meeting were read and approved. Committee on Revision of the Constitution determined not to submit a report, thinking the constitution would answer every purpose at the present time.

Dr. Hoff, of Ann Arbor, chairman of the Dental Protective Association, said the Association was flourishing, work was being carried on with gratifying success. Dr. Crouse was anxious that the membership be enlarged. It was not its object to confine its work to patents only, but along other lines, viz: influencing manufacturers to make a better class of goods at a fair compensation; by buying patents and working in conjunction with manufacturers.

Dr. Hoff urged the necessity of all practitioners becoming members.

Dr. Harris, of Pontiac, member of the University Visiting Committee, could make no report.

Dr. Moore, of Detroit, offered the following resolution:

"*Resolved*, That the University Visiting and Consulting Committee be discontinued in this Association as being superfluous."

Letters from Dr. H. A. Smith, of Cincinnati, and Dr. A. H. Fuller, of St. Louis, thanking the society for donation to work on Pre-historic Crania, were received and ordered placed on file.

Dr. Dorrance, of Ann Arbor, in lieu of a paper described the different forms of Tray's impression materials and gave some valuable points in the use of the same in securing accurate impressions.

A communication from C. S. Case, of Jackson, stating his inability to be present was read, received, and ordered placed on file.

On motion of Dr. J. Lathrop, of Detroit, seconded by Dr. Harris, of Pontiac, Dr. L. G. Whiting, of Saginaw, and Dr. J. Robinson, of Jackson, were made honorary members of the Association.

The committee on a permanent place of meeting, through the chairman Dr. J. Taft, rendered the following report of progress, which was received and committee continued.

REPORT OF PROGRESS.

Your committee, to whom was referred the question of a home or fixed place for holding the annual meetings of this Association, would report:

That they have considered the subject, in its various aspects and bearings, and present the following *suggestions*, viz: that the next annual meeting be held at the Dental College in Ann Arbor, at a time to be fixed by the Executive Committee, not later than June 20th, 1893.

Also, that Ann Arbor be fixed as the permanent home of the society.

Also, that the Executive Committee, in connection with this Committee on place of meeting, or its successor, be requested to consider the propriety of making provision for a more extended series of clinics and demonstrations that may occupy from two to four days, and if, in the judgment of the joint committee, such a work seem practicable and desirable, the Committee have power to make preparation for such a course of work.—J. Taft, chairman.

REPORT OF CLINICS.

Dr. J. G. Walton, in his clinic, brings to our attention the advantages of half shields in those cases of abrasion where the teeth, being worn very short, or the patient being aged or infirm, he may have an operation that may be quickly done, and promise comfort and usefulness, often saving him late in life the struggle of learning to become expert with artificial teeth.

He makes no claim of an artistic operation as he performs it, but assures us that it is practical, and satisfactory to patient and dentist.

Dr. L. F. Owen, of Grand Rapids, gave a clinic on the different methods of root filling.

The doctor said he had no new methods to introduce, but filled roots as the occasion demands, and does not practice immediate root filling only in cases where the pulp is removed by mechanical means or by the use of cocaine.

In cases where the apical foramen is large, he fills that opening with gold, lead or tin, then fills with gutta-percha or cement.

Dr. W. H. Dorrance gave a clinic on porcelain inlays, describing simple methods, claiming not only its adaption, but its artistic beauty.

Dr. Gish of Jackson then read his paper on "Electricity as Applied to the Art and Science of Dentistry," which was discussed by the members present, stress being laid upon the therapeutic application.

Dr. Gish was commended for his efforts along this line. It was the sentiment of the meeting that he should continue investigating this work as he was so well fitted for it.

The meeting adjourned to 9 o'clock Saturday morning.

In the evening the Association was the invited guest of the dentists of Saginaw, at the beautiful Hotel Vincent. The following account of the proceedings is taken from the Saginaw Courier-Herald.

THE BANQUET.

The handsome dining room of the Hotel Vincent was last evening turned into a floral bower. The room was banked and filled with potted plants and palms; from every conceivable nook and corner hung festoons of roses and handsome vines. In the rear of the room Boos' superb orchestra was stationed. Some 18 small tables were set for the guests; at each place a buttonhole bouquet was to be found and one of the handsomest menu cards ever offered to a banqueted party. Thus arrangements were made for the banquet tendered by "The Dentists of Saginaw" to "The Michigan Dental Association." At 8:30 the guests were seated and partook of the palatable repast which the management of the Hotel Vincent had prepared with great care, and which consisted of the following

MENU:

New York Counts, Broiled Whitefish, Saratoga Chips, Beef Tenderloin, Mushrooms, Spring Chicken, Water Cress, Fried Frog Legs, New Potatoes, Vienna Rolls, Tea Biscuit, Plain Bread, Assorted Cake, Strawberries, Fruit, Crackers and Cheese, Tea and Coffee.

During the discussion of the eatables the orchestra rendered several selections and all were placed in a good humor. At the end of the last course the Arion quartette, composed of Messrs. Evans, Watrous, Bostwick and Mearns, rendered "Hark! The Trumpet Calleth," in such an acceptable manner that the dentists demanded another song, when they sang "Who Built the Ark?" which pleased the audience immensely.

President Corns then arose and in a brief speech returned thanks for the elegant supper and for the courteous treatment which they had all received from the management of the Hotel Vincent. He then called upon Rev. George F. Warren, who spoke a few words, and who was followed by Dr. J. Taft, Dean of the Michigan University. Dr. Taft responded to the toast "The Progress of our Profession." He said: "The progress that has been made in the dental profession can only be realized by those who have taken part in the great work." He cited the time when the blacksmith or the cobbler could extract a tooth as well as a dentist, and when a dentist was considered but little better than a thief, when the medical profession looked upon the dental profession as a miserable art. He then showed how at the present day they rank high in the professional walks of life and how the medical fraternity invite dentists to become members of their associations. In conclusion he said: "The progress has been upward and onward, and as the responsibility has increased so has the profession advanced to meet all requirements."

He was followed by Dr. N. S. Hoff, of Ann Arbor, who responded to the toast, "The Young Members." He entreated the young men of the profession not to give up their studies on the completion of their college course; nor to develop their business ability to the detriment of their professional ability. His remarks furnished much food for thought for the young men of the profession, and were well received.

Dr. George L. Field, of Detroit, responded to the toast "Our Lady Patients." The doctor soon had his listeners in a roar of laughter and kept them in good humor throughout his remarks.

Dr. A. T. Metcalf, of Kalamazoo, responded to the toast "Our Saginaw Friends." The doctor did not confine himself much to his subject, but he told a number of good anecdotes which enlivened the dentists.

Dr. J. A. Robinson, of Jackson, was then called upon. Dr. Robinson, is one of, if not the oldest practicing dentist in the United States, being 80 years of age, and having been in active practice for the last 57 years. The old gentleman is as hale and hearty as can be, and his short talk was of great interest to all. He told of his early practice and the contempt he was held in. His talk was bright and wholesome throughout and showed that the doctor is a great lover of his profession.

The quartette then sang "Good night," after which the Orchestra played "Auld Lang Syne," and the banquet was brought to a close by all singing the old familiar song.

---

SATURDAY MORNING, JUNE 4th.

Meeting was called to order by the President. Minutes were read and approved.

Dr. Hoff read a paper on "Antizymotics, or Agents which Prevent Fermentation and Putrefaction."

Dr. Taft moved the discussion be postponed owing to shortness of time.

On motion of Dr. Field, seconded by Dr. Moore, the election of officers was ordered and the result was as follows:

For President, Dr. N. S. Hoff, of Ann Arbor.

For 1st Vice-President, Dr. W. P. Morgan, of Saginaw.

For 2d Vice-President, Dr. J. L. Sweetnam, of Manistee.

For Secretary, Dr. J. Ward House, of Grand Rapids.

For Treasurer, Dr. George H. Mosher, of Jackson.

The following names were reported favorably by the Board of Censors, and, on motion which was carried, were elected members of the Association:

F. S. Anderson, of Midland; F. H. Essig, of Dowagiac; William S. Milligan, of Saginaw; J. S. Wilde, of Petoskey; W. H. Kessler, of Detroit; A. T. Loeffler, of Saginaw; J. D. Shunck, of West Bay City; W. P. Morgan, of Saginaw; R. G. Porter, of Petoskey; George S. Root, of Grand Rapids; Manning

A. Birge, of Grand Rapids; H. O. Harvey, of Battle Creek; J. A. White, of Saginaw; William Gooding, of Saginaw; Irwin Meyers, of Saginaw; L. G. Dean, of Vassar; J. F. Perry, of Muskegon; F. E. Benton, of Fenton; W. B. Flynn, of West Branch; R. W. Sweetnam, of Muskegon; Dr. Lamb, of——; J. A. Fritz, of Cass City; Gordon W. Welch, of Jackson; W. H. Knapp, of Jackson.

Dr. Field presented the following resolution, which was ordered engrossed on the minutes and printed in the daily papers:

"*Resolved*, that the thanks of this Association be tendered to our Brother Dentists of this city for their kind and brotherly reception tendered us during this meeting.

To the Hotel Vincent, from the proprietor down through every grade of employee till we pass the bar keeper, for their very kind and courteous treatment during our stay.

To the Mayor and City Council for the efforts put forth by them for our general good and comfort during our brief stay in this the beautiful City of Saginaw.

To those who have added to the general benefit and interest of this meeting by what they contributed as clinics and essays.

To our very worthy friend, Mr. Calkins, and his worthy lieutenant, Mr. Barnett, for all that they have done at this time that has added so much to the general happiness of this meeting.

And to Divine Providence for the most delightful weather that we have enjoyed whilst here, as we can now all go home with the assurance that the dry spell is broken.

Also to the Press of this city, for their very correct and satisfactory reports of our meeting."

The President appointed the following members of the different committees:

Local Committee on Arrangements, Dr. W. H. Jackson, of Ann Arbor; Dr. L. P. Hall, of Ann Arbor.

Member of the Executive Committee, Dr. H. C. Corns.

Member of the Board of Censors, Dr. W. A. Dorland, of Grand Rapids.

Member University Visiting Committee, Dr. W. Williams, Sault Ste Marie.

Supervisor of the Clinic in Operative Dentistry, Dr. J. A. Watling. of Ypsilanti.

Supervisor of the Clinic in Prosthetic Dentistry, Dr. W. H. Dorrance of Ann Arbor.

Moved by Dr. Corns, seconded by Dr. Field, that an order for $5.00 be drawn to pay the janitor; the following bills be ordered paid: Dean Printing Company, $6.25; Secretary, $15.83.

The Treasurer's report was read as follows:

Cash on hand at the beginning of present session, $162.09; receipts at present session, $174.00; expenses present session, $52.07; leaving a balance of $284.02.

Moved by Dr. Field, seconded by Dr. Moore, that we adjourn to meet at Ann Arbor not later than June 20th.

Meeting adjourned.

